# Overall Survival of Papillary Thyroid Carcinoma Patients: A Single-Institution Long-Term Follow-Up of 5897 Patients

**DOI:** 10.1007/s00268-018-4479-z

**Published:** 2018-01-18

**Authors:** Yasuhiro Ito, Akira Miyauchi, Minoru Kihara, Mitsuhiro Fukushima, Takuya Higashiyama, Akihiro Miya

**Affiliations:** Department of Surgery, Kuma Hospital, 8-2-35 Shimoyamate-dori, Chuo-ku, Kobe, Hyogo 650-0011 Japan

## Abstract

**Introduction:**

Papillary thyroid carcinoma (PTC) generally shows an excellent prognosis except in cases with aggressive backgrounds or clinicopathological features. Although the cause-specific survival (CSS) of PTC patients has been extensively investigated, the overall survival (OS) of these patients is unclear. We herein investigated both the OS and CSS of a large PTC patient series.

**Materials and methods:**

We enrolled 5897 PTC patients who underwent initial surgery between 1987 and 2005 (658 males and 5339 females; median age 51 years). Their median postoperative follow-up period was 177 months. Univariate and multivariate analyses for OS and CSS assessed the effects of gender, older age (≥55 years), distant metastasis at diagnosis (*M*1), significant extrathyroid extension, tumor size (cutoffs 2 and 4 cm), large node metastasis (*N* ≥ 3 cm), and extranodal tumor extension.

**Results:**

To date, 387 patients (7%) in this series have died from various causes, including 117 (2%) due to PTC. The 10-, 15-, and 20-year OS rates are 97, 95, and 90%, respectively. Older age and *M*1 were important prognostic factors for OS and CSS. Older age was a more significant factor than *M*1 for OS and vice versa for CSS. In the older patients, *M*1 was a prominent prognostic factor for both OS and CSS. In the young patients, *M*1 had less prognostic impact than in the older patients, and the prognostic values of *M*1 and *N* ≥ 3 cm for OS and CSS were identical and similar, respectively.

**Conclusions:**

The most important prognostic value for OS was patient age, indicating that PTC is generally indolent. However, the control of distant metastasis in older patients remains a future challenge in order to further improve their OS and CSS. PTC of ≥3 cm in young patients should be carefully followed, even in the absence of metastases, and these patients should undergo aggressive therapies for recurrent lesions and metastases.

## Introduction

Papillary thyroid carcinoma (PTC) is the most common malignancy arising from thyroid follicular cells. Although PTC frequently metastasizes to the regional lymph nodes, it generally has an excellent prognosis. There are, however, several clinicopathological and background features that predict a poor prognosis. Some of these features—namely older age, distant metastasis, lymph node metastasis, extrathyroid extension, tumor size, and completeness of resection—have been adopted in staging systems such as the AMES, MACIS, and AJCC TNM classifications [1–3]. Our institution has also studied prognostic factors of PTC and found that older age (≥55 years), clinical node metastasis (*N*1), distant metastasis (*M*1) at diagnosis, significant extrathyroid extension (Ex) on intraoperative findings, extranodal tumor extension (LN-Ex) on intraoperative findings, and large tumor size (*T*) significantly affected the prognosis of patients [4, 5].

Several end points are used to evaluate the prognostic significance of the various clinicopathological features of human carcinomas, usually disease-free survival (DFS), cause-specific survival (CSS), and overall survival (OS). However, since differentiated thyroid carcinomas are generally indolent and the patients show excellent prognoses, long-term follow-ups are mandatory to evaluate prognostic factors based on OS. Therefore, although the DFS and CSS of PTC patients have been extensively studied, little is known about the OS of these patients. In this study, we investigated the OS and the clinicopathological features affecting the OS in a large series of PTC patients undergoing long-term follow-up.

## Patients and methods

### Patients

We enrolled 5897 patients who underwent an initial surgery between 1987 and 2005 at Kuma Hospital: 658 males and 5339 females with an age range of 7–89 years (median 51 years). The postoperative follow-up period ranged from 4 to 357 months (median 177 months). Our exclusion criteria were (1) patients who had undergone only local palliative surgery and (2) patients who had other malignancies arising from the thyroid such as follicular carcinoma, medullary carcinoma, anaplastic carcinoma, and malignant lymphoma.

### Thyroidectomy

The surgical approaches in the era when the enrolled patients underwent initial surgery were significantly different from those in use now. Total thyroidectomy was performed only when carcinoma lesions were located in both lobes and when physicians considered the cases to have aggressive characteristics. *M*1 patients underwent total thyroidectomy in order to undergo radioactive iodine (RAI) therapy. In our series, total and near total (residual thyroid ≤1 g) thyroidectomy were performed in 2956 and 89 patients, respectively. Subtotal thyroidectomy and limited thyroidectomy (such as hemithyroidectomy and isthmectomy) were performed in 536 and 2316 patients, respectively.

### Lymph node dissection

Central node dissection was almost routinely performed, regardless of whether it was therapeutic or prophylactic, except for cases that were not clearly diagnosed as PTC on preoperative cytology. In this series, 5619 patients (95%) underwent central node dissection. One thousand and thirty-four patients were diagnosed as having *N*1b, and all of them underwent therapeutic modified radical neck dissection (MND). Prophylactic MND was performed in 3433 of 4863 *N*0 or *N*1a patients (71%). In the past, Japanese institutions, including ours, almost routinely performed prophylactic MND, because regional node metastasis was difficult to evaluate on imaging studies. Ultrasound was introduced in the latter half of the 1980s and was found to be helpful to preoperatively diagnose node metastasis. However, in the era when our enrolled patients underwent surgery, many patients still received prophylactic MND. Whether or not each patient underwent prophylactic MND was decided based on the surgeons’ discretion, but tumors with large size, extrathyroid extension, or *N*1a more frequently underwent prophylactic MND [6]. In other words, patients were normally recommended prophylactic MND unless their PTCs were regarded as early stage by surgeons. This is because the diagnostic power of ultrasound for lymph node metastasis was not strong in the early phase. The diagnostic accuracy of ultrasound improved in stages, and in 2006, we finally limited the indications for prophylactic MND to patients having large tumors and significant extrathyroid extension; a decade later, support for this decision was provided by a study showing that prophylactic MND only for large tumors with significant extrathyroid extension could improve the lymph node recurrence rates [6]. In the present series, 13 of our patients had also undergone mediastinal node dissection, because metastasis to the mediastinal node was suspected on imaging studies.

### Postoperative scintigraphy and RAI therapy

Postoperative scintigraphy using a small amount of RAI (≤13 mCi) was performed in 972 patients. Only 83 patients underwent postoperative RAI ablation (≥50 mCi). Forty-seven of the 68 patients who had distant metastasis at surgery had undergone RAI therapy (≥100 mCi) after total thyroidectomy. None of the patients underwent RAI therapy to control localized neck disease. Nineteen of the 47 patients underwent RAI scintigraphy to investigate whether the RAI uptake was positive. Over the time range of this series, RAI scintigraphy was frequently performed for PTC patients at the physicians’ discretion, mainly in cases with aggressive features such as a large number of node metastases, significant extrathyroid extension, and aggressive histology.

### Postoperative follow-up

All of the patients were followed by blood examinations (thyroid-stimulating hormone, thyroglobulin and anti-thyroglobulin antibody) and imaging studies such as ultrasound once or twice per year. Chest roentogenography, CT scan, and bone scintigraphy were also used for follow-up at the physicians’ discretion. We regarded cases as having PTC recurrence when recurrent lesions were detected by imaging studies. Lymph node recurrence was diagnosed on cytology, and thyroglobulin (Tg) measurement was performed for suspicious nodes using the washout from the fine needle aspiration biopsy (FNAB) [7]. Postoperative high or elevated Tg levels without structural evidence of recurrence on imaging studies were not judged as having recurrence, because we do not regard such cases as candidates for any treatment. We obtained informed consent to participate from all patients in advance of the postoperative follow-up; all patients agreed to be followed up by questionnaire even after leaving the hospital.

### Statistical analyses

We used the Kaplan–Meier method with log-rank tests (univariate analysis) and the cox proportional hazard model (multivariate analysis) for the statistical analyses, which were performed using the software program StatView. *p* values <0.05 were accepted as significant, and *p* values between 0.1 and 0.05 were regarded as having marginal significance.

## Results

### Clinicopathological features of the enrolled patients

Table 1 summarizes the clinicopathological features of the 5897 patients enrolled in this study. To date, 606 (10%) have experienced recurrence to local lesions. Recurrence to the remnant thyroid, soft tissue, and regional lymph nodes was observed in 60, 42, and 531 patients, respectively. Twenty-seven patients showed recurrence in two local lesions. One hundred and thirty-two (2%) have experienced recurrence to distant organs such as the lungs, bone, and brain. Seventy-nine have had recurrences to both local lesions and distant organs. To date, 387 patients (7%) have died of various causes, and of these, 117 (2%) died due to PTC.

**Table 1:** Clinicopathological features of the 5897 patients

Variable	No. of patients (%)
Age	
≥55 years	2355 (40)
<55 years	3542 (60)
Gender	
Male	676 (11)
Female	5221 (89)
*M* factor	
*M*1	68 (1)
*M*0	5829 (99)
Extrathyroid extension	
Yes	781 (13)
No	5116 (87)
Tumor size	
>4 cm	595 (10)
2.1–4 cm	1909 (32)
≤2 cm	3393 (58)
*N* factor	
*N*1 (>3 cm)	202 (3)
*N*1 (≤3 cm)	969 (16)
*N*0	4726 (81)
Extranodal tumor extension	
Yes	145 (2)
No	2752 (98)
Recurrence at local lesion(s)	
Yes	606 (10)
No	5291 (90)
Recurrence at distant organ(s)	
Yes	132 (2)
No	5765 (98)
Death due to thyroid carcinoma	
Yes	117 (2)
No	5780 (98)
Death from a cause other than thyroid carcinoma	
Yes	269 (5)
No	5628 (95)

### OS and CSS for the patient series

**Fig. 1:**
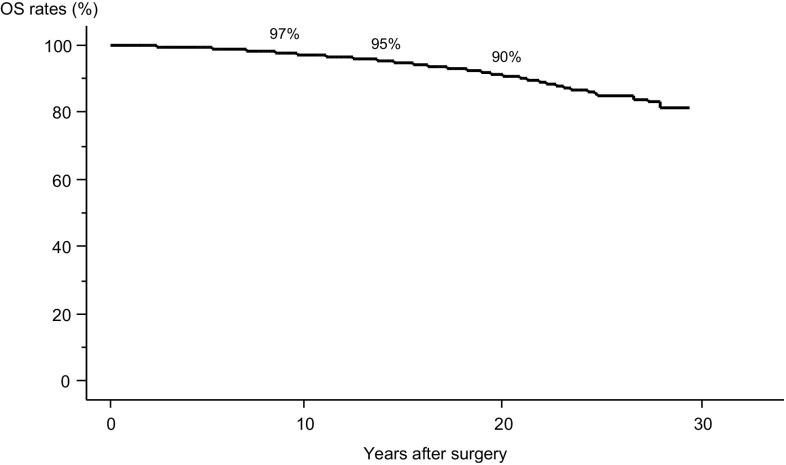
Kaplan–Meier curve of OS in the entire series of patients

Figure 1 presents the Kaplan–Meier curve for the OS of the patients; the 10-, 15-, and 20-year OS rates were 97, 95, and 90%, respectively. Table 2 demonstrates the univariate and multivariate analyses of variables for OS. We adopted the following clinicopathological features and background characteristics as variables: (1) male gender, (2) older age (≥55 years), (3) distant metastasis at diagnosis (*M*1), (4) extrathyroid extension (Ex), (5) tumor size >4 cm and 2.1–4 cm, (6) clinical node metastasis (*N*) ≥3.0 cm and <3.0 cm, and (7) extranodal tumor extension (LN-Ex). All of these were regarded as having significant prognostic values in our previous studies [4]. *N* ≥ 3 cm was initially identified as having strong prognostic value by Sugitani et al. [8]. Ex is defined as extrathyroid extension corresponding to T4 in the AJCC classification system, but is evaluated on intraoperative findings. LN-Ex indicates carcinoma extension requiring the dissection of organs adjacent to the metastatic nodes, such as recurrent laryngeal nerve, jugular vein, trachea, vagal nerve, and phrenic nerve. This was also evaluated based on intraoperative findings. Ex and LN-Ex were based on gross findings [4]. As a result, *M*1, older age, male gender, Ex, *T* > 4 cm, *N*1 and LN-Ex were found to have prognostic value for OS both in univariate and multivariate analyses. In the multivariate analysis, older age and *M*1 in particular had much higher hazard ratios (HRs) than the other variables, and older age had greater multivariate impact than *M*1 (the HRs for older age and *M*1 were 8.33 and 6.06, respectively).

**Table 2:** Univariate and multivariate analyses of variables for OS in a 5897 PTC patient series

Variable	Univariate	Multivariate	
	*p* value	*p* value	HR (95% CI)
Male gender	<0.0001	0.0104	1.42 (1.08–1.85)
Age > 55 years	<0.0001	<0.0001	8.33 (6.49–10.75)
Distant metastasis (*M*1)	<0.0001	<0.0001	6.06 (3.92–9.35)
Extrathyroid extension (Ex)	<0.0001	<0.0001	2.22 (1.74–2.82)
Tumor size (*T*)			
>4.0 cm	<0.0001	<0.0001	2.27 (1.71–3.00)
2.1–4.0 cm	0.8425	0.7321	1.04 (0.82–1.33)
Clinical node metastasis (*N*1)			
>3.0 cm	<0.0001	<0.0001	2.64 (1.81–3.85)
<3.0 cm	<0.0001	0.0025	1.52 (1.16–1.99)
Extranodal tumor extension (LN-Ex)	<0.0001	0.0073	1.75 (1.16–2.64)

**Fig. 2:**
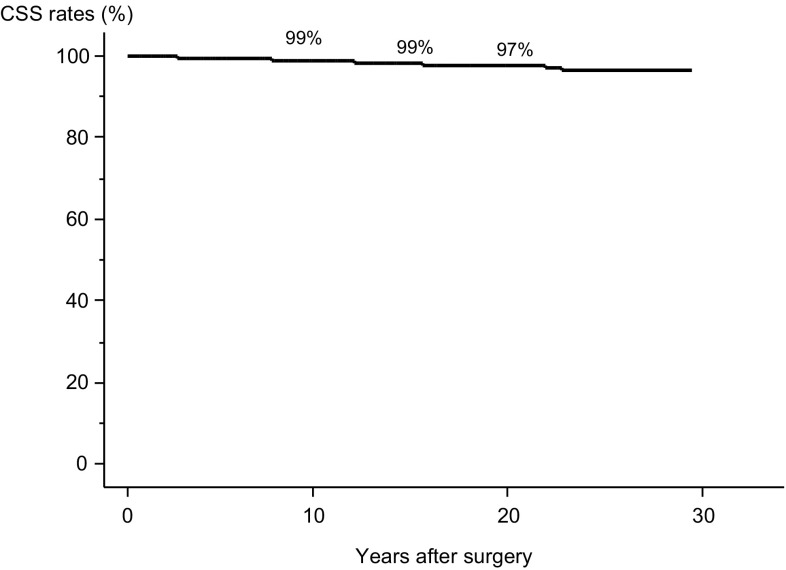
Kaplan–Meier curve of CSS in the entire series of patients

The Kaplan–Meier curves for the CSS of the patients are shown in Fig. 2. The 10-, 15-, and 20-year CSS rates were 99, 99, and 97%, respectively. Table 3 summarizes the results of the univariate and multivariate analyses for the CSS of the entire series of patients. All enrolled factors significantly or marginally affected the CSS of patients on univariate analysis. In multivariate analysis, *M*1, older age and *T* > 4 cm had greater prognostic value than the others. Unlike in the OS analysis, *M*1 had greater prognostic value for CSS (HR 10.41) than either older age or *T* > 4 cm (HRs 7.46 and 7.14, respectively).

**Table 3:** Univariate and multivariate analyses of variables for CSS in a 5897 PTC patient series

Variable	Univariate	Multivariate	
	*p* value	*p* value	HR (95% CI)
Male gender	<0.0001	0.0572	1.52 (0.99–2.34)
Age > 55 years	<0.0001	<0.0001	7.46 (4.63–12.05)
Distant metastasis (*M*1)	<0.0001	<0.0001	10.41 (6.06–17.86)
Extrathyroid extension (Ex)	<0.0001	<0.0001	4.08 (2.62–6.33)
Tumor size (*T*)			
>4.0 cm	<0.0001	<0.0001	7.14 (4.10–12.35)
2.1–4.0 cm	0.0565	0.0175	1.96 (1.12–3.40)
Clinical node metastasis (*N*1)			
>3.0 cm	<0.0001	<0.0001	3.28 (1.86–5.78)
<3.0 cm	0.0004	0.0295	1.71 (1.18–2.78)
Extranodal tumor extension (LN-Ex)	<0.0001	0.0001	3.12 (1.75–5.56)

### OS and CSS of the older (≥55 years) and younger (<55 years) patients

Patient age is a very important prognostic factor for PTC patients, especially with respect to carcinoma-related death, indicating that the biological characteristics of PTC depend significantly on patient age. We therefore analyzed the OS and CSS of the older (≥55 years) and younger patients (<55 years) in our series. In the univariate analysis for the subset of older patients, all factors enrolled significantly affected the OS of patients (Table 4). In the multivariate analysis for older patients, *M*1 was the most prominent prognostic factor (HR 8.00 vs 1.55–2.78 for the other factors). Similarly, as shown in Table 5, all factors affected CSS in the univariate analysis for this subgroup, and in multivariate analysis, all factors except for male gender independently reflected CSS. The HR for *M*1 (13.33) was higher than that of other variables.

**Table 4:** Univariate and multivariate analysis of OS variables in the 2355 PTC patients >55 years

Variable	Univariate	Multivariate	
	*p* value	*p* value	HR (95% CI)
Male gender	0.0002	0.1506	1.26 (0.92–1.72)
Distant metastasis (*M*1)	<0.0001	<0.0001	8.00 (4.81–13.16)
Extrathyroid extension (Ex)	<0.0001	<0.0001	2.13 (1.62–2.80)
Tumor size (*T*)			
>4.0 cm	<0.0001	<0.0001	2.78 (2.24–3.85)
2.1–4.0 cm	0.0321	0.2681	1.17 (0.88–1.56)
Clinical node metastasis (*N*1)			
>3.0 cm	<0.0001	0.0006	2.24 (1.41–3.85)
<3.0 cm	<0.0001	0.0045	1.55 (1.15–2.10)
Extranodal tumor extension (LN-Ex)	<0.0001	0.1010	1.51 (0.92–2.51)

**Table 5:** Univariate and multivariate analysis of CSS variables in the 2355 PTC patients >55 years

Variable	Univariate	Multivariate	
	*p* value	*p* value	HR (95% CI)
Male gender	0.0004	0.2859	1.32 (0.79–2.22)
Distant metastasis (*M*1)	<0.0001	<0.0001	13.33 (6.99–25.64)
Extrathyroid extension (Ex)	<0.0001	<0.0001	3.86 (2.30–6.49)
Tumor size (*T*)			
>4.0 cm	<0.0001	<0.0001	9.80 (5.08–19.23)
2.1–4.0 cm	0.0078	0.0119	2.36 (1.21-4.63)
Clinical node metastasis (*N*1)			
>3.0 cm	<0.0001	0.0115	2.43 (1.22–4.83)
<3.0 cm	<0.0001	0.0446	1.72 (1.01–2.90)
Extranodal tumor extension (LN-Ex)	<0.0001	0.0250	2.90 (1.13–5.78)

In the subset of younger patients, male gender, *M*1, Ex, *T* > 4 cm, *N* ≥ 3 cm and LN-Ex significantly and *N* < 3 cm marginally affected the OS of patients in univariate analysis. In multivariate analysis, male gender, *M*1, *N* ≥ 3 cm, and LN-Ex independently and Ex marginally affected OS. The HRs of *M*1 and *N* ≥ 3 cm were almost identical (4.02 and 4.01) and higher than those for the other factors (Table 6). As shown in Table 7, all factors except for T 2.1–4.0 cm and *N* < 3 cm univariately affected CSS. In the multivariate analysis, *M*1, Ex, *N* ≥ 3 cm and LN-Ex were recognized as independent prognostic factors. The HRs of *M*1 (10.75) and *N* ≥ 3 cm (7.69) were prominently high. In contrast, the HRs of Ex and LN-Ex were much lower, at 3.32 and 4.55, respectively.

**Table 6:** Univariate and multivariate analysis of OS variables in the 3542 PTC patients <55 years

Variable	Univariate	Multivariate	
	*p* value	*p* value	HR (95% CI)
Male gender	<0.0001	0.0260	1.87 (1.01–3.25)
Distant metastasis (*M*1)	<0.0001	<0.0001	4.02 (1.71–10.53)
Extrathyroid extension (Ex)	< 0.0001	0.0621	1.74 (0.97–3.11)
Tumor size (*T*)			
>4.0 cm	0.0270	0.7339	1.11 (0.61–2.02)
2.1–4.0 cm	0.2898	0.1528	0.69 (0.42–1.15)
Clinical node metastasis (*N*1)			
>3.0 cm	<0.0001	<0.0001	4.01 (2.01–7.81)
<3.0 cm	0.0750	0.1128	1.63 (0.89–2.99)
Extranodal tumor extension (LN-Ex)	<0.0001	0.0260	2.86 (1.34–6.10)

**Table 7:** Univariate and multivariate analyses of CSS variables in the 3542 PTC patients <55 years

Variable	Univariate	Multivariate	
	*p* value	*p* value	HR (95% CI)
Male gender	<0.0001	0.2052	1.83 (0.72–4.65)
Distant metastasis (*M*1)	<0.0001	<0.0001	10.75 (3.38–33.33)
Extrathyroid extension (Ex)	<0.0001	0.0110	3.32 (1.32–8.47)
Tumor size (*T*)			
>4.0 cm	0.0004	0.2073	2.00 (0.68–5.88)
2.1–4.0 cm	0.7825	0.8460	1.11 (0.40–3.10)
Clinical node metastasis (*N*1)			
>3.0 cm	<0.0001	0.0003	7.69 (2.53–23.26)
<3.0 cm	0.4260	0.3414	1.85 (0.52–6.58)
Extranodal tumor extension (LN-Ex)	<0.0001	0.0078	4.55 (1.49–13.89)

## Discussion

We investigated the OS and CSS of a large number of PTC patients who underwent long-term follow-up after surgery (median 177 months). We demonstrated that (1) older age and *M*1 significantly affected both OS and CSS; (2) in the subset of patients ≥55 years, M1 was the prominent prognostic factor not only in CSS but also OS; and (3) in patients <55 years, *M*1 strongly affected OS and CSS, but the prognostic value of *N* ≥ 3 cm was equal to that of *M*1 for OS.

To date, several studies employing large numbers of patients have investigated the prognostic significance of patient age. In recent studies, 45 years has generally been adopted as the cutoff age [9–11], although some studies have demonstrated that other cutoffs, such as age 50 or 60 years, were also prognostic [8, 12, 13]. We previously demonstrated that a cutoff age of 55 years more significantly affected the DFS and CSS of PTC patients than other cutoffs [14], and thus we also adopted this cutoff in this study.

In our present study, older age (≥55 years) was the strongest prognostic factor for OS. This was clearly because the number of patients who died of PTC was small, indicating that most PTCs generally have an indolent character. However, in the subset analysis of older patients, *M*1 was the prominent factor affecting both their OS and CSS. We previously demonstrated that old age was a strong predictor of carcinoma death for *M*1 patients and patients after the appearance of distant recurrence [15, 16]. Taken together, these findings suggest that distant metastasis/recurrence strongly shortens the life span of old patients. There are some conventional strategies for distant metastasis/recurrence, such as reoperation, RAI therapy and extrabeam radiotherapy, but controlling distant metastasis/recurrence by these modalities is often difficult and new therapeutic strategies are expected. Recently, tyrosine kinase inhibitors such as sorafenib and lenvatinib became available for differentiated thyroid carcinoma with RAI-refractory metastases, due to the prolonged progression-free survival of patients treated with these agents [17, 18]. One phase III study demonstrated that lenvatinib significantly improved the OS of the subset of patients older than 65 years (median age 71 years) [19], suggesting that this is one of the promising therapeutic strategies for PTC recurrence in older patients.

*M*1 also strongly affected the CSS and OS of younger patients, but their HRs were much smaller than those of the older patients, and thus *M*1 was less prognostic in this group. The prognostic significance of large *N* was the same as that of *M*1 for OS and similar to that of *M*1 for CSS. As indicated above, large *N* was originally proposed as a prognostic factor by Sugitani et al. [8]. Also, we revealed that large *N* (≥3 cm) independently affected the CSS of young patients without metastasis (*M*0 patients) [4]. These findings indicate that, although PTC in young patients is generally indolent, not only *M*1 patients but also patients with large *N* should be carefully followed postoperatively, even if they could undergo locally curative surgery and do not have distant metastasis at surgery.

Our study has some limitations. This is a retrospective study and patients underwent therapies that are significantly different from those used now. As indicated in the Patients and Methods section, in our series, 71% of patients underwent not only central node dissection but also prophylactic MND, which is significantly different from our surgical strategy at present. Recently, we studied the significance of prophylactic MND for *N*0–1a PTC patients [20]. We showed that prophylactic MND did not improve the lymph node recurrence-free and distant recurrence-free survivals of these patients, although lymph node recurrence-free survival was improved in patients having PTC larger than 3 cm with significant extrathyroid extension. We can therefore speculate that prophylactic MND for *N*0–1a*M*0 PTC is not likely to have a major impact on the prognosis of patients, including CSS and OS. In addition, although the percentage of patients who underwent RAI scintigraphy in our series was high, only 83 patients received RAI ablation. Also, the indications for total thyroidectomy were very different from those in use today. Although it is unlikely that these issues had a major effect on the OS or CSS of patients, it is important to bear in mind that these therapeutic strategies were significantly different from the standard therapies used today, not only in Western countries but also in Japan. All these factors should be considered limitations of this study.

In this study, we showed that older age was the most important prognostic factor for OS, indicating that PTC is generally indolent and overtreatment should be avoided in many cases. However, we also showed that *M*1 was the strongest prognostic factor not only for CSS but also for OS of older patients, indicating that controlling distant metastasis/recurrence in older patients is very important and should be pursued as a future goal. In contrast, most PTCs in young patients show an excellent prognosis, but clinicians should be aware that even in young *M*0 patients, PTCs with large *N* require extensive initial surgery, careful postoperative follow-up, and immediate and aggressive therapies for recurrent lesions.
